# Supplementary material for: Deep learning-based segmentation of lithium-ion battery microstructures enhanced by artificially generated electrodes
Source: Nat Commun. 2021 Oct 27;12:6205. doi: 10.1038/s41467-021-26480-9 (PMC8551326; doi:10.1038/s41467-021-26480-9)
Supplement: Supplementary file 1 — Supplementary Information [file 41467_2021_26480_MOESM1_ESM.pdf]

Deep Learning-based Segmentation of Lithium-Ion Battery Microstructures enhanced by artificially generated Electrodes

Supplementary Information

Simon Müller[1, †], Christina Sauter[1, †], Ramesh Shunmugasundaram[1], Nils Wenzler[1], Vincent De Andrade[2], Francesco De Carlo[2], Ender Konukoglu[1] & Vanessa Wood[1*]

[1] Department of Information Technology and Electrical Engineering, ETH Zurich, Switzerland

[2]Advanced Photon Source, Argonne National Laboratory, USA

† The authors contributed equally to the work

* Corresponding author

Author contact information:

Simon Müller
Gloriastrasse 35 ETZ H67
8092 Zurich, Switzerland
+41 (0)44 633 8986
simmuell@ife.ee.ethz.ch

Christina Sauter
Gloriastrasse 35 ETZ H67
8092 Zurich, Switzerland
+41 (0)44 632 603
sauterc@ife.ee.ethz.ch

Ramesh Shunmugasundaram
Gloriastrasse 35 ETZ H67
8092 Zurich, Switzerland
+41 (0)44 632 6469
ramesh.s@ife.ee.ethz.ch

Nils Wenzler
Gloriastrasse 35 ETZ H65
8092 Zurich, Switzerland
+41 (0)44 632 5816
wenzlern@ethz.ch

Vincent De Andrade
Advanced Photon Source
Argonne National Laboratory
IL 60439, USA
+1 630 252 9241
vdeandrade@aps.anl.gov

Francesco De Carlo
Advanced Photon Source
Argonne National Laboratory
IL 60439, USA
+1 630 252 0148
decarlo@aps.anl.gov

Ender Konukoglu
Sternwartstrasse 7
8092 Zürich Switzerland
+41 (0)44 633 8816
kender@vision.ee.ethz.ch

*Vanessa Wood
Gloriastrasse 35 ETZ J86
8092 Zurich, Switzerland
+41 (0)44 632 6654
vwood@ethz.ch

Note 1. Electrochemical Experiment

Three electrodes are cycled galvanostatically in a half-cell configuration. The voltage vs. capacity curves for cells cycled 2, 5 and 8 cycles (Supplementary Fig. 1a, b and c respectively) over a potential range of 10 mV – 1.5V at a rate of C/20 with VMP3 battery cycling system (Supplementary Fig. 1d). The curves show progressive capacity fade with increasing cycle number. Each protocol ends with a 20 h period of constant voltage (3 V) to ensure delithiation of the graphite-silicon electrode under examination.

Taking the derivative of the capacity with respect to the potential for the cell operated for one cell cycled eight times reveals clear peaks that correspond to features associated with (de)lithiation of graphite (solid lines) and silicon (dashed lines) (Supplementary Fig. 1e) [1]. Due to the low volume fraction of silicon and its proximity to a pronounced feature in graphite, the peak at 0.08V commonly reported for silicon [2], [3] cannot be clearly identified. While the intensity peaks associated with graphite remain relatively constant over cycling, the magnitude of the peaks associated with silicon peaks decrease (e.g., see the peak associated with lithiation of silicon around 0.23 V shown in Supplementary Fig. 1f), indicating that the capacity fade comes from silicon.

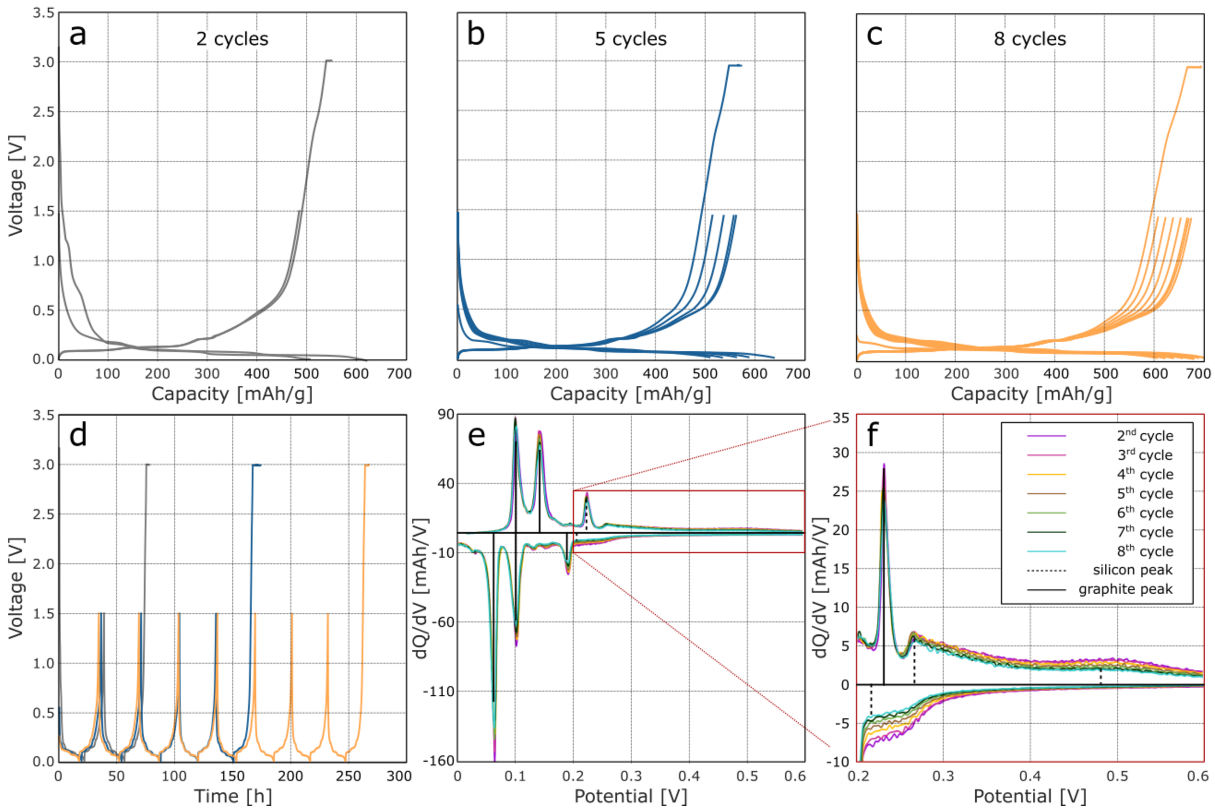

Supplementary Fig. 1: *Capacity vs. voltage profile for samples cycled twice (a), five times (b), and eight times (c) according to the cycling protocol shown in (d). The derivative of the capacity with respect to the voltage is shown in (e) and in more detail in (f) for potentials between 0.2 V and 0.6 V. The black solid lines indicate the features stemming from (de)lithiation of graphite while the dashed lines associated indicate the features stemming from (de)lithiation with silicon.*

Note 2. Fourier Shell Correlation

The spatial resolution is calculated for each sample by means of the Fourier shell correlation. As a threshold, the half-bit criterion is used [4]. Supplementary Table 1 shows the resolution values for all samples. The average spatial resolution is 119 nm.

Supplementary Table 1: *Spatial resolution based on the Fourier shell correlation for all imaged samples.*

	Pristine	2 Cycles	5 Cycles	8 Cycles
Sample 1	110.98 nm	114.62 nm	125.00 nm	125.00 nm
Sample 2	109.43 nm	103.57 nm	118.85 nm	118.37 nm
Sample 3	129.46 nm	142.86 nm	121.85 nm	113.28nm

Note 3. Synthetic Structures

Synthetic structures are generated in two steps. First, a basic structure is created (Supplementary Fig. 2a and b) and, second, this basic structure is given the features of a real structure (Supplementary Fig. 2c and d) using a style transfer determined by the CycleGAN algorithm [5], [6]. The synthetic structures (Supplementary Fig. 2e and f) have the macroscopic structure defined by the basic structure but a higher and more realistic degree of detail (compare Supplementary Fig. 2b and f). Further details in the following sections.

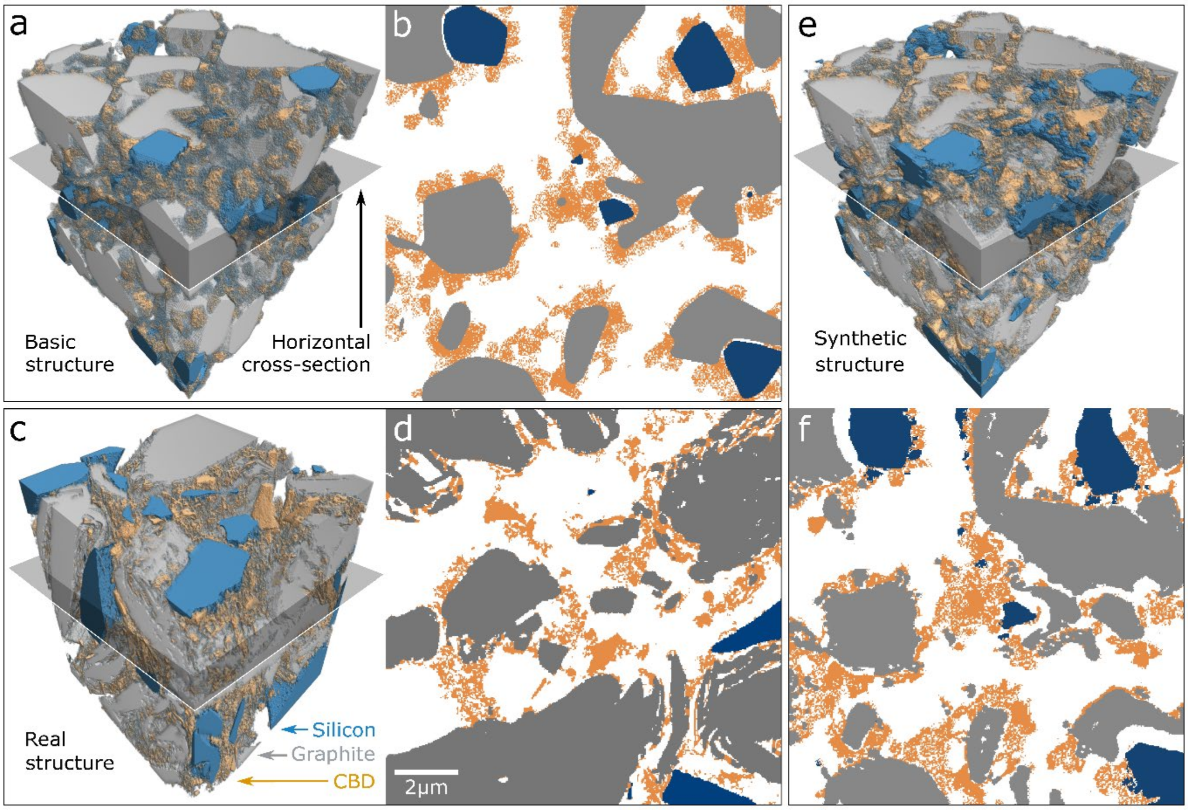

Supplementary Fig. 2: *Starting from a basic computer-generated structure (a and b), features of high-resolution segmented structures (c and d) are used to create realistic synthetic structures (e and f).*

Note 3.1. Basic structure

The basic structure (Supplementary Fig. 2a and b and Supplementary Fig. 3d) consists of four material phases: graphite (Supplementary Fig. 3a), silicon (Supplementary Fig. 3b) and carbon black-binder domain (CBD) (Supplementary Fig. 3c).

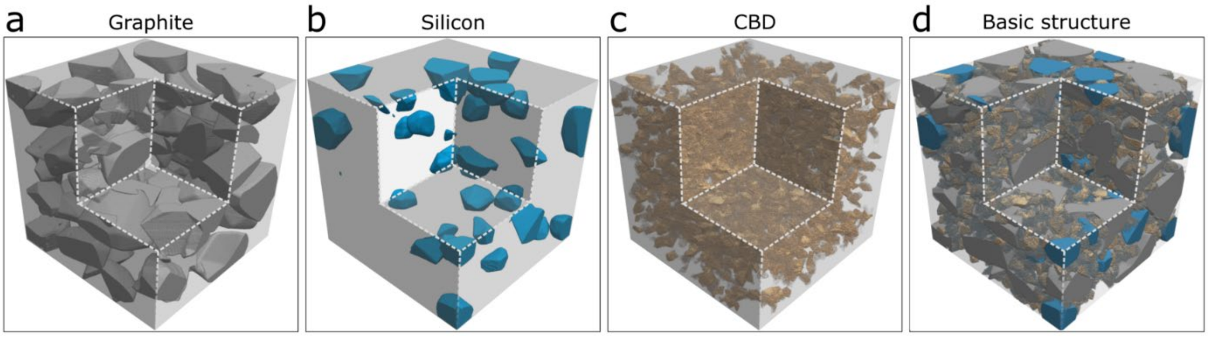

Supplementary Fig. 3: *The basic structure is generated starting with the graphite phase (a), adding the silicon phase (b), and finally the CBD (c). The combination results in the basic structure (d).*

The smallest entity of each phase is the particle. A particle generator written in MATLAB creates a convex particle based on a specified number of edge points (Supplementary Fig. 4a and f). The larger the number of edge points, the more spherical the particle. The distance of each edge point to the origin (i.e. the particle center) can be defined as well as the maximum and minimum directional components (x, y, and z, see Supplementary Fig. 4b and e). These options allow the creation of various particle shapes and sizes. The larger the distances the bigger the particles and the more similar the directional components, the more spherical the particles. To achieve non-convex particles (Supplementary Fig. 4f and h), a convex particle is simply subtracted from another one. In a further step, the particle is bent onto a two-dimensional polynomial function (Supplementary Fig. 4d and g), smoothed and finally rotated in space (Supplementary Fig. 4c and h).

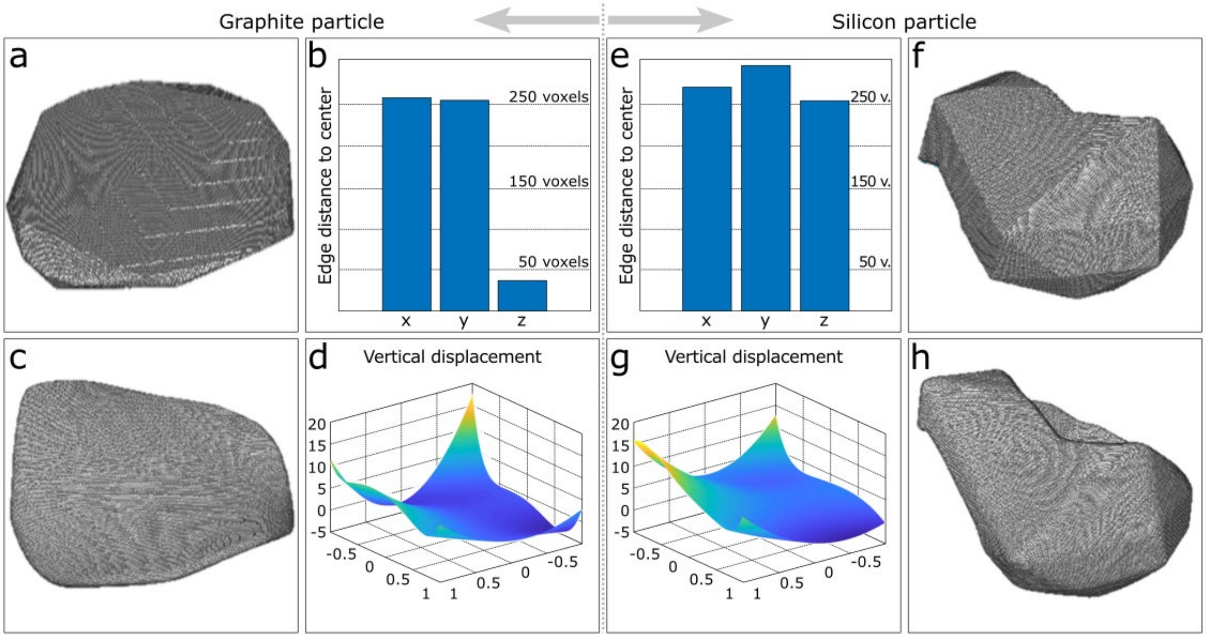

Supplementary Fig. 4: *Particle generator for graphite (a-d) and silicon particles (e-h) including generation of a raw particle (a and f) with characteristic anisotropy (b and e). The raw particle is warped using a 2D polynomial (d and g) and finally smoothed (c and h).*

Graphite particles are large flake-like particles (i.e., they have anisotropic directional components) that, due to electrode manufacturing, tend to be oriented parallel to the x-y plane. Silicon particles are smaller, more isotropic, and randomly oriented. To create the carbon black-binder domain (CBD), polygons are generated like for graphite and silicon. The characteristic fluffiness is added by subjecting the CBD particles to an internal porosity, which corresponds to a value randomly chosen between 5% and 40% (only used for the "basic structure").

All the particles are assembled into a structure such that the user defined volume fractions are taken into account. First, the graphite particles are placed in the previously empty volume. Second, the silicon particles are placed among the graphite particles. The degree of overlap between particles of a given phase or between particles of two different phases can be set depending on the user's preferences. Here, we allow overlap of graphite particles with one another to create random, more complex structures, but we allow no overlap for graphite and silicon because we do not observe this in real structures. In case there is not enough space to fulfill the criterion given by the user-defined volume fractions, smaller and smaller silicon particles are created. Finally, the CBD is added to the graphite and silicon phase with no overlap allowed.

Note 3.2. Style transfer

The goal of a style transfer is to find characteristic features of the different phases in real structures and map them onto the basic, computer-generated structure so as to obtain synthetic structures that are useful for training a neural network.

The style transform performed using a CycleGAN algorithm [5] according to Forro et al. [6]. It requires training, which can be given by providing unpaired computer-generated basic structures and real structures. The (discrete) segmented real structures and distance maps (i.e., a continuous descriptor instead of the discrete representation) of the basic structures are used to allow for better convergence of the CycleGAN algorithm.

The basic structure (Supplementary Fig. 5a) contains the desired macroscopic properties (volume fractions, preferred orientation, and anisotropy of particles). It is converted into a distance map (Supplementary Fig. 5b). Segmented electrode structures from a previous study combining transmission x-ray tomographic microscopy and ptychographic x-ray computed tomography [7] are used as the real data (Supplementary Fig. 5d). The CycleGAN algorithm then generates a realistically looking synthetic structures (Supplementary Fig. 5c) from the distance map (Supplementary Fig. 5b) of the basic structure (Supplementary Fig. 5a) and a distance map (Supplementary Fig. 5e) of the real structure (Supplementary Fig. 5d), which is not further used.

The CycleGAN algorithm is trained on 1000 distance maps of computer-generated structures and 1000 distance maps of real 256x256 pixel images for 200 epochs. Different models were trained (varying the volume fractions present in the real structures, graphite 30%-40%, silicon 5%-20%, CBD 5%-15%) and the six visually most appealing ones were selected to translate the basic structures into realistic synthetic structures (Supplementary Fig. 5c). While the exact number of chosen models (six) is completely arbitrary, the idea behind using a variety of models for the style transfer is to introduce a larger variance amongst the resulting synthetic structures.

The CycleGAN algorithm works with two-dimensional images. In order to apply it on the three-dimensional basic structures, these are cut into cross-sections parallel to the in-plane orientation (x-y plane). The CycleGAN style transfer is run on the cross-sections, which are later on assembled to the full image stack. Three-dimensional consistency is reached by median-filtering the silicon and graphite phase with a 1D filter kernel pointing along the through-plane direction (z).

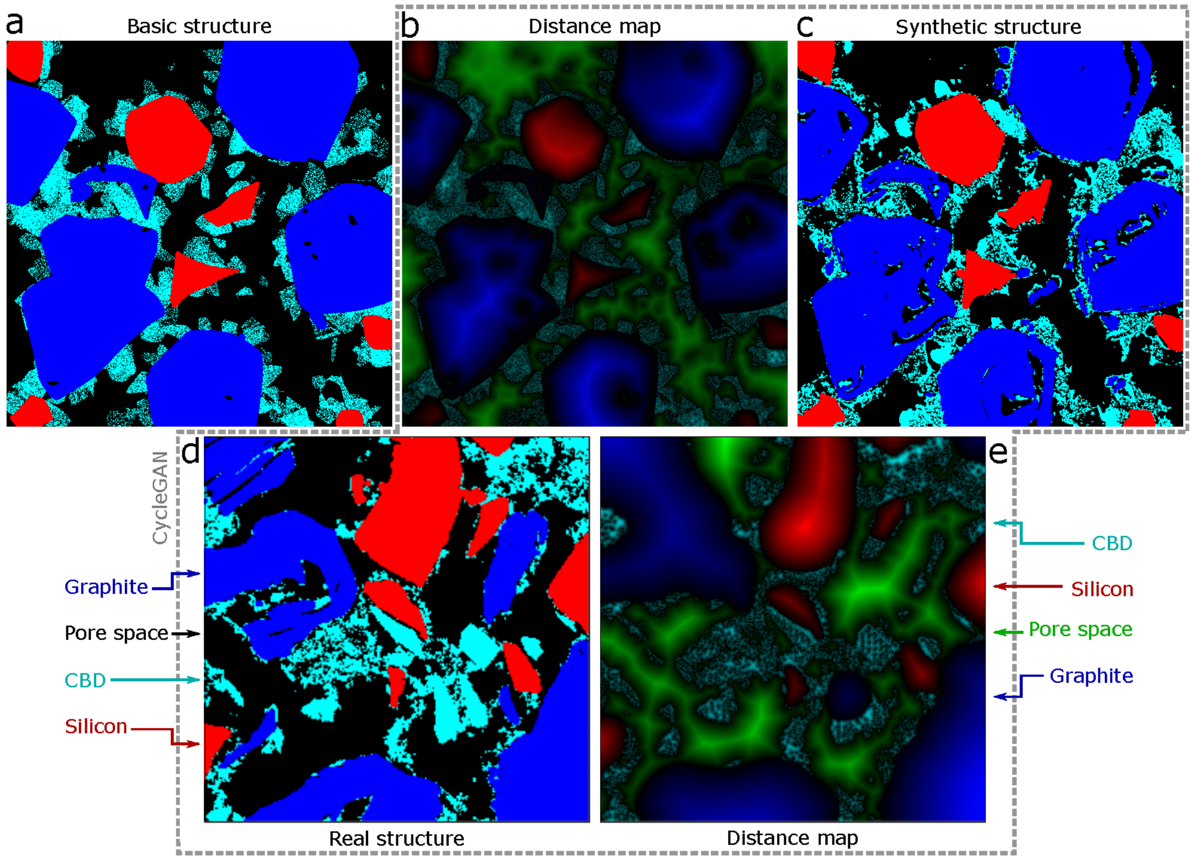

Supplementary Fig. 5: *A style transfer on an example cross-section. The basic structure (a) is converted to a distance map (b). The CycleGAN algorithm then starts with the unpaired image data (b and d) and applies the style of the real structure onto the distance map (resulting in a synthetic structures (c)) and the style of the distance map onto the real structure (resulting in a "fake distance map" (e) that is not used further on).*

Note 4. Tomography Simulation

Refractive indexes (β for absorption and δ for phase contrast) are taken from henke.lbl.gov [8] and assigned to the corresponding material phases of the synthetic structure such that we have β and δ maps of the synthetic structures.

From the β map, 1210 projections are taken between 0 and 180 degrees using the ASTRA toolbox [9].

The delta map is first smoothed and then the first and second order gradients are taken along the incident direction of the virtual x-rays and the projections of the first and second order gradient are calculated. Then the delta map is rotated by 180/1210 degrees, the gradients are taken along the new incident direction of the virtual x-rays and the projections are calculated. This procedure is carried out for each of the 1210 projections.

As a result, we have three sets of projections (β, first gradient of δ and second order gradient of δ) that are combined by the means of a weighted sum (ratio of $1{:}1\mathrm{x}10^{-6}{:}1\mathrm{x}10^{-4}$) to a final set of projections. The final set of projection is reconstructed using the filtered back projection algorithm in the ASTRA toolbox. In order to get realistic simulation results, the gray value histogram is shifted, stretched, and compressed, and zero mean Gaussian noise is added to match the histogram of real tomography images.

When comparing simulated tomography to real tomography images, we reach a structural similarity index (SSIM) [10] of approximately 0.28. Split up into its components, we find rather high values for the accordance in luminance (≈0.92) and contrast (≈0.94), and low values (≈0.32) for the similarity in structure. The SSIM value however is not used as a target function but to get an overall idea of how to adjust the gray value histogram.

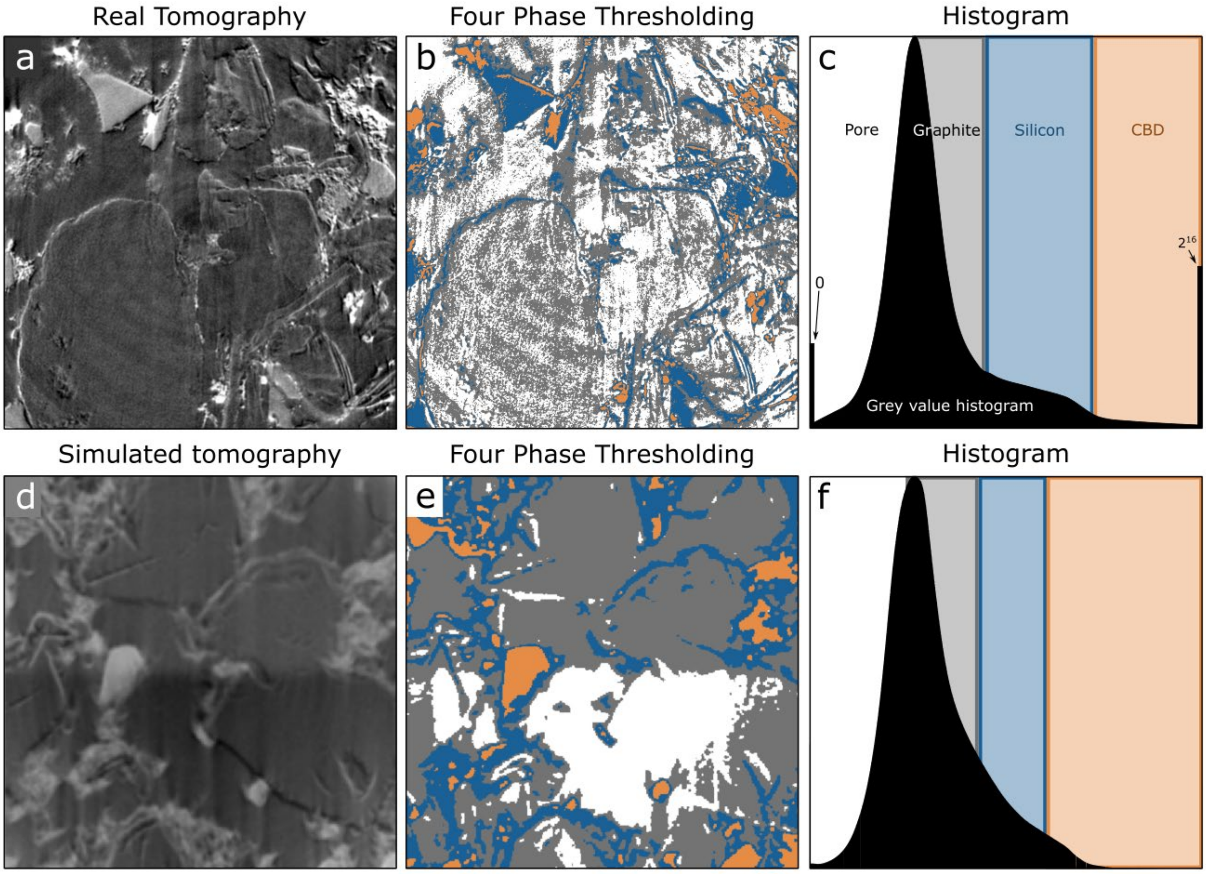

Supplementary Fig. 6: *Cross-section of a real tomography image (a), the segmentation applying a k-means algorithm (b), and the corresponding gray value histogram with the four material phases indicated (c). A slice of a simulated tomography image (d), the k-means segmentation (e), and the gray value histogram (f).*

Looking at the simulated tomography (Supplementary Fig. 6d) it can be seen that the resolution is lower compared to the real tomography data (Supplementary Fig. 6a). This is due to the fact that the simulated projections are three times smaller in each dimension (width and height of the virtual detector) resulting in a nine times smaller dataset, which is necessary for time-efficient simulation. It is also noticeable that the simulated tomography is less noisy. This is not representative for all simulated images but serves to increase the variation in noise and blur in the training dataset. Furthermore, the gray values of silicon particles and CBD are closer in the simulated case than in the real tomography.

As for the real case, thresholding with a k-means algorithm does not lead to satisfying results for the simulated tomography and the problems are similar to the ones found for the real tomography: mistaking pore space for graphite, assigning particle boundaries to the silicon phase and local illumination differences that make thresholding extremely difficult.

Comparing the gray value histograms of real and simulated tomography (Supplementary Fig. 6c and f) shows an overall correspondence with the main peaks lining up. It can be noticed however that the shoulder assigned to silicon is higher and narrower in the simulated case compared to the real tomography images. Also, there are less saturated pixels. As saturated pixels are mostly located at the top and bottom of the sample and likely to be cut off, this should not present an issue.

Overall, the simulated tomography images match the reality and, most importantly, represent the same difficulties regarding segmentation. Naturally, there is room for improvement as the applied simulation can be seen as a first order approximation to the much more complex wave propagation approach that ideally should be used to simulate phase contrast effects that are of importance for light materials as graphite and carbon black. It has also to be noted that aside from the physics of the tomography the influence of the detector (e.g. saturation limits, normalization) has to be taken into account to ensure comparability.

Note 5. Manual Segmentation

Three-dimensional manual segmentation is performed with the Dragonfly software on one of the pristine datasets. The different features of interest are identified by the human eye and painted according to the specific material phase.

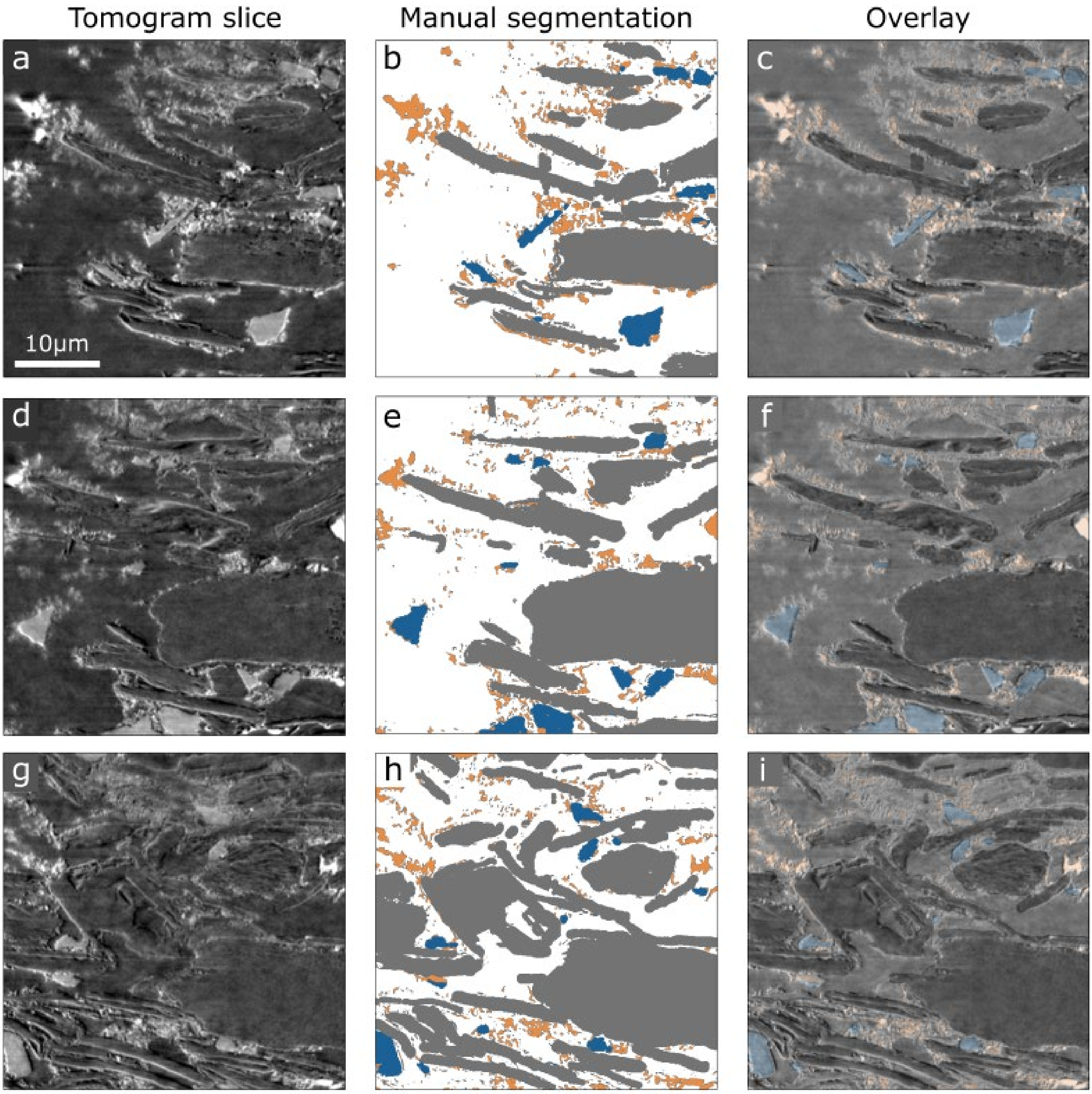

Supplementary Fig. 7: *Three cross-sections of the manually segmented tomogram. The raw data is shown in panels (a), (d), and (g), the segmented slices are displayed in (b), (e), and (h). Panels (c), (f), and (i) show overlays of the raw data and the segmentation.*

Note 6. Deployment

Each volumetric dataset to be segmented is 768x768x768 voxel in size. We choose to run the neural network on subvolumes of 48x256x256 voxel in order to (i) comply with the memory limitations and to (ii) provide an excerpt of image data that is likely to include particle boundaries in the 256x256-plane while still containing enough context in the third dimension. Thus, the volumetric dataset image is split into subvolumes. Since the subvolume dimensions are not isotropic, the 768x768x768 voxel image can be sampled in three ways: along the x-axis (with sub-volume dimensions of 48x256x256 voxels), along the y-axis (with sub-volume dimensions of 256x48x256 voxels), and along the z-axis (with sub-volume dimensions of 256x256x48 voxels). Sampling is done such that neighboring patches overlay by half their edge-length. This leads to 775 ($(3+2)^2 \times (16+15)$) sub-volumes per sampling direction.

All subvolumes of each sampling direction are evaluated by the neural network and returned as 4D softmax maps of dimension 48x256x256x4. While the first three dimensions have to be permuted according to the sampling direction, the fourth dimension holds the prediction values for the four phases (pore space, graphite, silicon, and CBD). The sum along the fourth dimension results to one for each voxel since it must belong to some phase.

We assume that the predictions in the center of the prediction volume are of higher confidence than the predictions at the border of the volume because of the larger number of neighboring voxel and thus larger amount of inferable information. Therefore, we multiply the softmax maps with a weighting matrix that is one in the center and radially decreases to 0.1 at the volume boundaries.

The weighted softmax maps are then assembled to an entire 768x768x768x4 softmax map. Due to the overlap in sampling, multiple predictions may exist for one voxel. These are simply added up. Note that due to the weighting and adding up of multiple predictions, the sum along the fourth dimension of the assembled softmax matrix no longer has to be one. The final prediction is achieved by taking the arguments of the maxima (argmax) of the sum of the three (one for each sampling direction) probability maps.

Note 7. Influence of Training Data

Because training the neural network on the labelled real data, which is available only to a limited amount, leads to unsatisfactory results (Supplementary Fig. 8b) complementary synthetic data is generated. It is specifically designed to add more variation in illumination, noise, and blur as these properties can vary amongst samples of different diameters (the thicker the sample, the more absorbing it is, the higher signal to noise it has) and samples imaged at different times (as the flux at the beamline is not constant over time). However, the synthetic data is not designed to train the network on its own as its variation is too extreme without real data used to "calibrate" the network model. As a consequence, a neural network trained on synthetic data exclusively performs poorly (Supplementary Fig. 8c). Only combining synthetic data with real data, thus resulting in a hybrid training dataset, leads to a significantly improved performance (see Supplementary Fig. 8d).

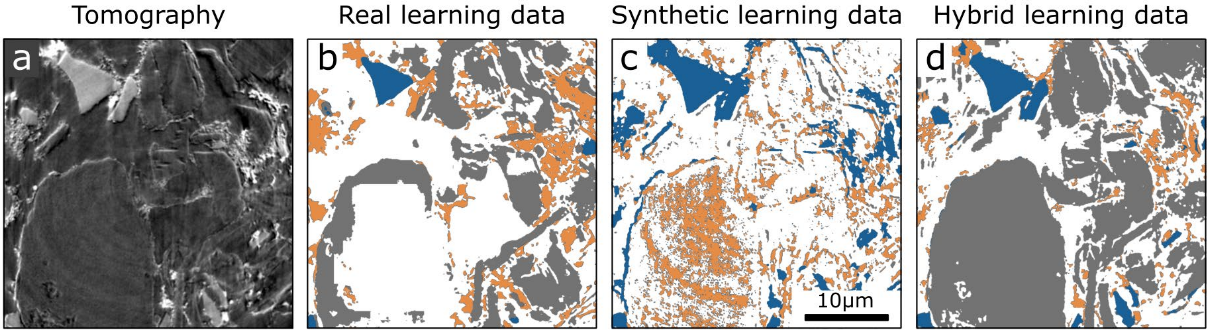

Supplementary Fig. 8: *Tomography cross-section (a) with corresponding segmentation stemming from a neural network trained on real learning data (b), synthetic learning data (c), or hybrid learning data (d).*

Potentially, one could also generate synthetic data that is very close to the real data and then aim at a neural network that is trained without any real data at all. However, generating synthetic data that looks realistically also requires real data for comparison, which would bring up the question why not to use this real data also to train the neural network.

Note 8. Segmentation Evaluation

In the main text the dice coefficient is used to evaluate and compare the segmentations resulting from a neural network trained on (i) real learning data only, (ii) hybrid (real and synthetic) learning data, and (iii) hybrid learning data with subsequent thresholding to the manual segmentation serving as "ground truth". In addition to the dice coefficient however, there exist various other metrics. We refer to Taha et al. [11] for a detailed description. To get the full picture, all calculated metrics can be found in Supplementary Table 2. It shows the calculated values for the different phases (pore space, graphite, silicon, CBD), the average, and the weighted average (by volume fractions of the different phases based on the manually segmented volume). The volume size is 500x400x576 voxels.

It can be noted that the introduction of synthetic learning data leading to hybrid learning data benefits the segmentation of all phases with respect to almost all metrics. The subsequent thresholding of the CBD leads to further improvement of the CBD and the pore space. Therefore, the average and the weighted average for all metrics are best for the hybrid learning data with subsequent thresholding.

Supplementary Table 2: *Segmentation evaluation measures.*

Metrics	Pore Space			Graphite			Silicon			CBD			Average			Weighted Average		
	Real learn. data	*Hybrid learn. data*	*Hybrid learn. data + thresh.*	*Real learn. data*	*Hybrid learn. data*	*Hybrid learn. data + thresh.*	*Real learn. data*	*Hybrid learn. data*	*Hybrid learn. data + thresh.*	*Real learn. data*	*Hybrid learn. data*	*Hybrid learn. data + thresh.*	*Real learn. data*	*Hybrid learn. data*	*Hybrid learn. data + thresh.*	*Real learn. data*	*Hybrid learn. data*	*Hybrid learn. data + thresh.*
Similarity																		
Dice Coefficient	0.626	0.692	0.715	0.645	0.769	0.769	0.688	0.816	0.816	0.381	0.582	0.719	0.585	0.715	0.755	0.626	0.727	0.744
Jaccard Coefficient	0.456	0.529	0.557	0.476	0.624	0.624	0.525	0.689	0.689	0.235	0.411	0.561	0.423	0.563	0.608	0.457	0.573	0.593
Area under ROC Curve	0.654	0.735	0.754	0.702	0.773	0.773	0.772	0.892	0.892	0.865	0.842	0.906	0.749	0.811	0.831	0.690	0.763	0.775
Cohen Kappa	0.307	0.478	0.515	0.411	0.542	0.542	0.680	0.810	0.810	0.333	0.558	0.703	0.433	0.597	0.642	0.369	0.523	0.546
Rand Index	0.548	0.620	0.638	0.589	0.646	0.646	0.967	0.976	0.976	0.773	0.908	0.941	0.719	0.788	0.800	0.592	0.658	0.667
Adjusted Rand Index	0.097	0.240	0.276	0.178	0.292	0.292	0.668	0.799	0.799	0.279	0.528	0.679	0.305	0.465	0.512	0.163	0.297	0.320
Interclass Correlation	0.625	0.691	0.714	0.644	0.768	0.768	0.688	0.816	0.816	0.381	0.582	0.719	0.584	0.714	0.754	0.625	0.726	0.743
Volumetric Sim. Coeff.	0.982	0.905	0.914	0.852	0.941	0.941	0.740	0.965	0.965	0.443	0.807	0.860	0.754	0.905	0.920	0.888	0.919	0.926
Mutual Information	0.069	0.176	0.204	0.134	0.228	0.228	0.092	0.133	0.133	0.085	0.104	0.149	0.095	0.160	0.179	0.101	0.195	0.210
Distance																		
Hausdorff Distance**	82.80*	52.40*	52.40*	72.30*	75.60*	73.20*	258.6	156.1	156.1	56.80*	70.10	57.70	117.6*	88.60*	84.90*	82.60*	67.60*	65.90*
Average Hausdorff Dist.**	5.187*	1.349*	1.302*	3.288*	2.197*	1.531*	8.468	1.575	1.575	1.661*	1.331	0.806	4.700*	1.613*	1.303*	4.245*	1.725*	1.395*
Mahanabolis Dist.**	0.400	0.146	0.101	0.321	0.085	0.085	0.216	0.062	0.062	0.273*	0.358	0.080	0.234*	0.163*	0.082*	0.351*	0.125*	0.091*
Variation of Information	1.852	1.596	1.544	1.661	1.539	1.539	0.170	0.150	0.150	0.745	0.425	0.308	1.107	0.927	0.885	1.654	1.465	1.437
Global Consistency Error	0.569	0.434	0.409	0.475	0.405	0.405	0.027	0.023	0.023	0.167	0.078	0.053	0.309	0.235	0.222	0.488	0.390	0.377
Probabilistic Distance	0.002	0.002	0.002	0.002	0.001	0.001	0.002	0.001	0.001	0.006	0.003	0.002	0.003	0.002	0.001	0.002	0.002	0.001
Classic Measures																		
Sensitivity	0.638	0.631	0.659	0.562	0.817	0.817	0.546	0.789	0.789	0.861	0.722	0.836	0.652	0.740	0.775	0.610	0.728	0.745
Specificity	0.670	0.839	0.849	0.842	0.729	0.729	0.999	0.995	0.995	0.870	0.963	0.976	0.845	0.882	0.887	0.771	0.799	0.804
Precision (Confidence)	0.615	0.764	0.783	0.757	0.726	0.726	0.930	0.845	0.845	0.245	0.488	0.630	0.637	0.706	0.746	0.675	0.736	0.751
Accuracy	0.656	0.745	0.763	0.711	0.770	0.770	0.983	0.988	0.988	0.870	0.952	0.969	0.805	0.864	0.873	0.703	0.775	0.784

*due to memory limitations, the volume is split into four quarters (250x200x576 voxel each) and the metric is calculated independently on each quarter and then averaged.

**the value is reported in voxels, with 1 voxel=61 nm

Note 9. Additional Structural Analysis

Macroscopically, the tortuosity τ is related to the porosity ε of porous electrodes by the Bruggeman exponent α and has been studied for porous electrode microstructures [12].

$$\tau = \epsilon^{-\alpha}$$

The fact that the Bruggeman exponent calculated on the electrode structures with the CBD included is smaller than the exponent calculated on the structures neglecting the CBD (Supplementary Table 3) indicates that the increase in the tortuosity brought about by CBD does not only come from a decrease in porosity but that the CBD also causes further morphological changes.

Supplementary Table 3: *Bruggeman exponent calculated for the different microstructure including and neglecting the CBD.*

		Number of cycles			
		pristine	2	5	8
Bruggeman exponent α [-]	Including CBD	1.78 ± 0.24	1.67 ± 0.22	1.67 ± 0.11	1.77 ± 0.11
	Neglecting CBD	1.93 ± 0.36	1.77 ± 0.31	1.75 ± 0.15	1.92 ± 0.16

In line with a constant effective transport coefficient over cycling, the pore diameter does not change over cycling (Supplementary Fig. 9a) and the median pore diameter of all samples is 720 nm. The thickness of the carbon black-binder domain (CBD) also stays constant at around 300 nm over the eight cycles (Supplementary Fig. 9b). While we could imagine that there are microstructural changes internal to the carbon black-binder domain during cycling, this cannot be resolved.

To eliminate the possibility that the different volume fractions of the phases in the different samples make sample-to-sample comparison difficult, we look for trends with cycling in sub-volumes (220x220x220 voxels) of the electrodes with similar volume fraction of the different phases. Again, trends in the morphology of the CBD and the pore size distribution are not observed (Supplementary Fig. 9c and d).

We used the PoroDict toolbox of the GeoDict2020 Software (Math2Market GmbH, Kaiserslauten, Germany) and calculated the pore size distribution and the CBD thickness by granulometry with symmetric boundary conditions.

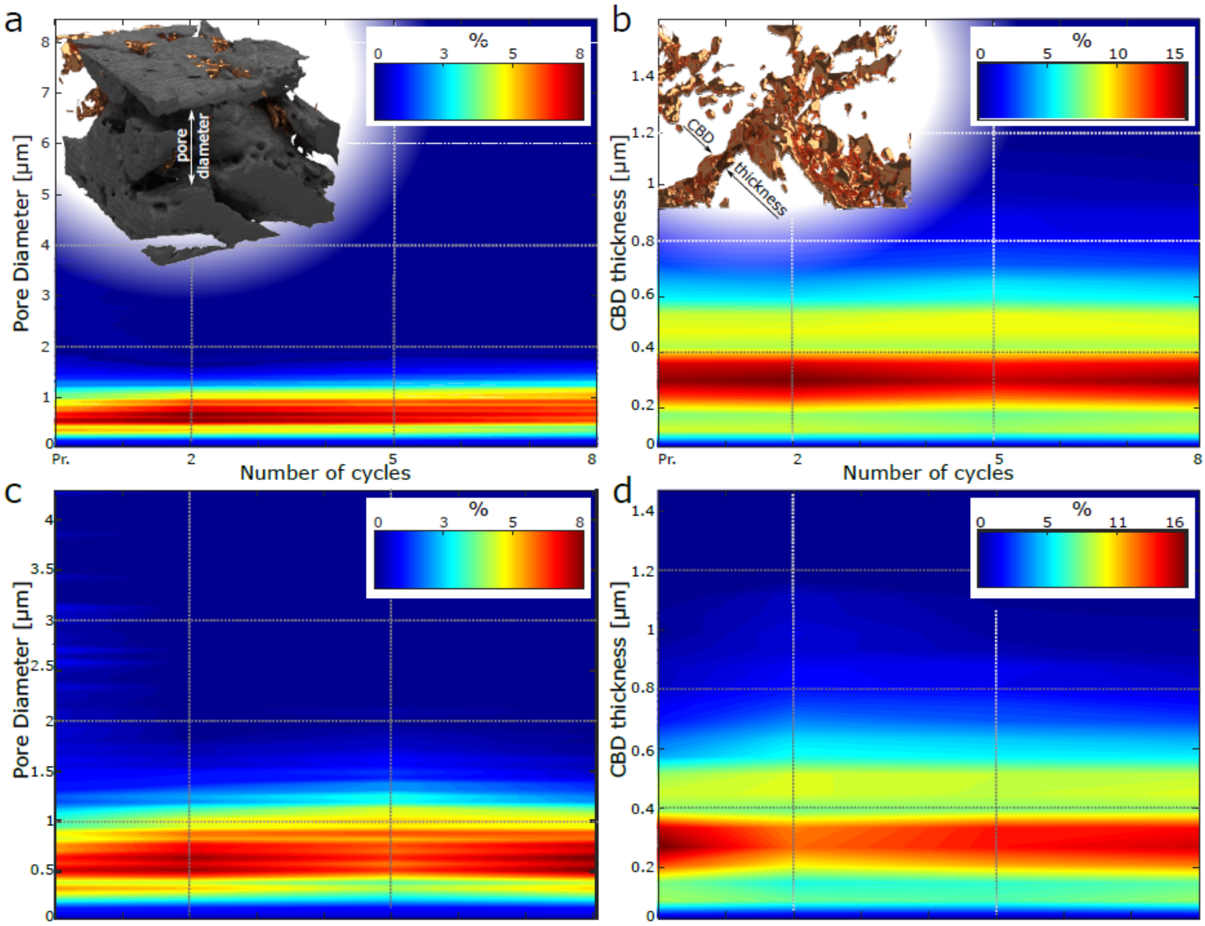

Supplementary Fig. 9: *Density plots of the geometric pore diameter distribution of the complete volume (a) and CBD thickness distribution of the complete volume (b) for different cycling states. Subsamples (220x220x220 voxel) with similar volume fractions are analyzed. The geometric pore diameter distribution (c) and the CBD thickness distribution for different cycling states do not show any trends.*

References

[1] A. Missyul, I. Bolshakov, and R. Shpanchenko, "XRD study of phase transformations in lithiated graphite anodes by Rietveld method," *Powder Diffr.*, vol. 32, no. S1, pp. S56–S62, 2017.

[2] L. J. Krause, T. Brandt, V. L. Chevrier, and L. D. Jensen, "Surface Area Increase of Silicon Alloys in Li-Ion Full Cells Measured by Isothermal Heat Flow Calorimetry," *J. Electrochem. Soc.*, vol. 164, no. 9, pp. A2277–A2282, 2017.

[3] M. Wetjen, S. Solchenbach, D. Pritzl, J. Hou, V. Tileli, and H. A. Gasteiger, "Morphological Changes of Silicon Nanoparticles and the Influence of Cutoff Potentials in Silicon-Graphite Electrodes," *J. Electrochem. Soc.*, vol. 165, no. 7, pp. A1503–A1514, 2018.

[4] M. Van Heel and M. Schatz, "Fourier shell correlation threshold criteria," *J. Struct. Biol.*, vol. 151, no. 3, pp. 250–262, 2005.

[5] J. Y. Zhu, T. Park, P. Isola, and A. A. Efros, "Unpaired Image-to-Image Translation Using Cycle-Consistent Adversarial Networks," *Proc. IEEE Int. Conf. Comput. Vis.*, vol. 2017-Octob, pp. 2242–2251, 2017.

[6] S. J. Ihle *et al.*, "Unsupervised data to content transformation with histogram-matching cycle-consistent generative adversarial networks," *Nat. Mach. Intell.*, 2019.

[7] S. Müller, M. Lippuner, M. Verezhak, V. De Andrade, F. De Carlo, and V. Wood, "Multimodal Nanoscale Tomographic Imaging for Battery Electrodes," *Adv. Energy Mater.*, vol. 1904119, pp. 1–8, 2020.

[8] B. L. Henke, E. M. Gullikson, and J. C. Davis, "X-Ray Interactions With Matter," *X-ray interactions: photoabsorption, scattering, transmission, and reflection*, 1993. [Online]. Available: http://henke.lbl.gov/.

[9] W. van Aarle *et al.*, "Fast and flexible X-ray tomography using the ASTRA toolbox," *Opt. Express*, vol. 24, no. 22, p. 25129, Oct. 2016.

[10] A. Horé and D. Ziou, "Image quality metrics: PSNR vs. SSIM," *Proc. - Int. Conf. Pattern Recognit.*, pp. 2366–2369, 2010.

[11] A. A. Taha and A. Hanbury, "Metrics for evaluating 3D medical image segmentation: Analysis, selection, and tool," *BMC Med. Imaging*, vol. 15, no. 1, 2015.

[12] M. Ebner, D. W. Chung, R. E. García, and V. Wood, "Tortuosity anisotropy in lithium-ion battery electrodes," *Adv. Energy Mater.*, vol. 4, no. 5, 2014.
